# Measuring impact of a quality improvement initiative on glaucoma clinic flow using an automated real-time locating system

**DOI:** 10.1186/s12886-022-02495-8

**Published:** 2022-06-28

**Authors:** John A. Musser, Juno Cho, Amy Cohn, Leslie M. Niziol, Dena Ballouz, David T. Burke, Paula Anne Newman-Casey

**Affiliations:** 1 Department of Ophthalmology & Visual Sciences, University of Michigan Medical School, 1000 Wall St, Ann Arbor, MI 48105 USA; 2 Department of Industrial and Operations Engineering, University of Michigan, Ann Arbor, MI USA; 3 Department of Human Genetics, University of Michigan, Ann Arbor, MI USA

**Keywords:** Automated time and motion study, Lean-analysis, Process time, Pre-post analysis

## Abstract

**Background:**

Lean methodology helps maximize value by reducing waste, first by defining what value and waste are in a system. In ophthalmology clinics, value is determined by the number of patients flowing through the clinic for a given time. We aimed to increase value using a lean-methodology guided policy change, then assessed its impact on clinic flow using an automated radiofrequency identification (RFID) based real-time locating system (RTLS).

**Methods:**

A total of 6813 clinical visits occurred at a single academic institution’s outpatient glaucoma clinic between January 5, 2018 to July 3, 2018. Over that period, 1589 patients comprising 1972 (29%) of visits were enrolled, with 1031 clinical visits occurring before and 941 visits after a policy change. The original policy was to refract all patients that improved with pinhole testing. The policy change was not to refract patients with a visual acuity ≥20/30 unless a specific request was made by the patient. Pre-post analysis of an automated time-motion study was conducted for the data collected 3 months before and 3 months after the policy change occurred on March 30, 2018. Changes to process and wait times were summarized using descriptive statistics and fitted to linear mixed regression models adjusting for appointment type, clinic volume, and daily clinic trends.

**Results:**

One thousand nine hundred twenty-three visits with 1588 patients were included in the analysis. Mean [SD] age was 65.9 [14.7] years and 892 [56.2%] were women. After the policy change, technician process time decreased by 2.9 min (*p* < 0.0001) while daily clinical patient volume increased from 51.9 ± 16.8 patients to 58.4 ± 17.4 patients (*p* < 0.038). No significant difference was found in total wait time (*p* = 0.18) or total visit time (*p* = 0.83).

**Conclusions:**

Real-time locating systems are effective at capturing clinical flow data and assessing clinical practice change initiatives. The refraction policy change was associated with reduced technician process time and overall the clinic was able to care for 7 more patients per day without significantly increasing patient wait time.

## Introduction

Lean analysis is a promising approach to improve the quality of healthcare. Originally used in the manufacturing sector to increase efficiency [1], lean analysis breaks complex processes into parts and identifies each part as either “value added” or “non-value added.” [2] Lean guided efforts create value for patients by maximizing the value-added parts while minimizing the non-value added parts.

However, quantifying the impact of an operations change has traditionally required a labor-intensive process of manually observing each individual step in the process [3–7]. As a result, large-scale time-and-motion studies in clinics can be extremely expensive and cost-prohibitive [7, 8]. With the 2009 Health Information Technology for Economic and Clinical Health (HITECH) Act and rapid adoption of electronic health records (EHR), many hoped that audit log data mining could unveil detailed real-world clinic workflow timing [9–12]. Unfortunately, EHR logs do not capture patient or provider presence. Physicians who talk to their patients before sitting down at the computer to type notes have delayed audit logs that, in effect, represent talk-time as wait time. Presently, it is not possible to measure absolute patient-centered time study data with the EHR alone [13, 14]. Furthermore, EHR data requires retrieval and processing after an encounter is finished, making rapid clinical optimization difficult [15, 16].

These limitations inherent to traditional and EHR guided time-and-motion studies highlight the need for an automated system that can detect patient and provider location. The system should also be capable of providing granular workflow data, including non-EHR captured activities, in real-time to allow for rapid clinical optimization [9, 17, 18]. In the Kellogg Eye Center glaucoma clinic, we designed and validated a real-time locating system (RTLS) that automatically conducts continuous time-and-motion studies to assess wait and process times [19]. The RTLS provides detailed real-time workflow information for EHR and non-EHR captured activities at a fraction of the cost of manual observation [19]. The described study had two objectives: 1) develop a lean-analysis guided policy change to improve clinical workflow, and 2) use the RTLS system to measure the impact of that policy change.

## Materials and methods

### Study institution

This study was conducted at the University of Michigan Kellogg Eye Center, a tertiary academic eye care center in Ann Arbor, Michigan. In 2018, 12 glaucoma specialists at the glaucoma clinic completed 14,642 out-patient visits. The study was approved by the University of Michigan Institutional Review Board as quality improvement research and followed all tenets of the Declaration of Helsinki. Patients gave verbal consent at check-in to participate in the study. This study was waived from having to obtain written informed consent by the University of Michigan Institutional Review Board as a quality improvement study.

### RTLS system development

The RTLS system development process is described in greater technical detail in a previous publication [19]. To summarize, the RTLS uses passive radiofrequency identification (RFID) tags and sensors to achieve high reliability and localization accuracy at a low cost [19].

The RTLS consisted of 23 integrated ultra-high frequency RFID readers (ThingMagic, Astra-Ex, Woburn, MA) arranged to cover clinical spaces. Consenting physicians, technicians, trainees, and patients were given passive RFID tags (Zebra Impinj Monza 4QT, Seattle, WA), each with unique identifiers. RFID readers scanned the environment twice per second to locate the tags.

A custom Java management application collected time and location data from patients and providers. The RFID reader raw data were processed and stored in an encrypted and Protected Health Information-secure database (MySQL). The RTLS data were validated by direct observation of patient encounters and by EHR audit logs from workstations located in individual rooms. Hidden Markov Modelling was used to reduce noise from the data. The Hidden Markov Modeling smoothed RFID system data had an accuracy of 80.6% for patient location and 79.1% for provider location compared to direct observations [19].

### Policy change development

Key stakeholders participated in a lean methodology training session led by a certified coach. The stakeholder team consisted of a glaucoma clinic physician, two medical students, an engineer, an ophthalmic technician, a scheduler, and a clinic manager. Following the principles of lean analysis, the team clarified the terms “value” and “waste” in the setting of an outpatient ophthalmology clinic. “Value” was defined as (1) the amount of time patients spent with a provider, and (2) the number of patients treated in the clinic per day. “Waste” was defined as the amount of time patients spent with no provider interaction, or wait time. The team created a value-stream visual diagram that incorporates every process in the clinic flow. The team then mapped where the “value” and the “waste” occurred and identified potential areas of improvement [20].

Through this collaborative process, the group identified the clinic’s refraction policy as a potential area for improvement. The original policy required refraction of all patients whose visual acuity improved with pinhole testing. In the proposed new policy, refraction was performed only on patients with a visual acuity worse than 20/30, unless a specific request was made by the patient.

### Policy change evaluation

**Fig. 1:**
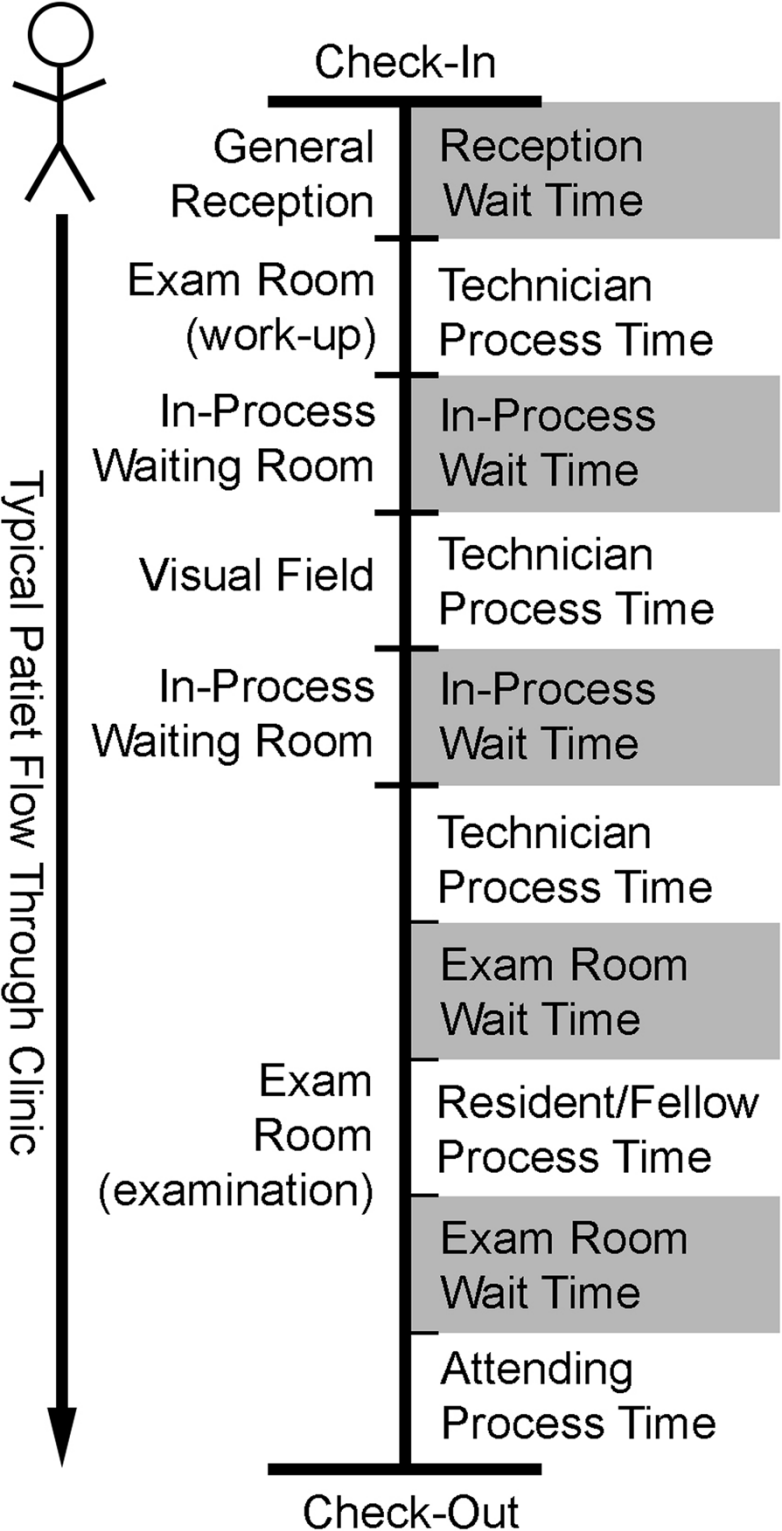
Timeline of typical glaucoma clinic visit from check-in to check-out, including process times (technician time, physician time, visual field time) and wait times (reception wait time, in-process wait time, exam room wait time)

We conducted a continuous time-and-motion study from January 5, 2018 to July 3, 2018. The RTLS collected data for 3 months before and 3 months after the policy change implementation on March 30, 2018. The time each patient spent at and between each step of their clinic visit was obtained (Fig. 1). When the RTLS detected a patient and a health care provider simultaneously in the same exam room, that time was labeled as “process time.” Process time was divided into three categories: 1) time spent with a physician in an exam room (including residents or fellows); 2) time spent with a technician in an exam room; 3) time spent on visual field testing. Wait time was divided into three location types: 1) the reception area, where the patient waits after checking in; 2) the in-process waiting area, where the patient waits for testing or for an exam room; and 3) the exam room, where the patient waits to be seen by a provider. When the RTLS detected a patient in one of the three location types without a health care provider, that time was determined to be “wait time.”

We queried the EHR records to obtain clinic volume data, visit types (e.g.*,* Laser Treatment, Post-Operative, New Patient, Return Visit, Urgent, and Visual Field Check), patient data (age, gender, race, ethnicity), and visit characteristics (day of week, appointment time).

### Statistical analysis

Descriptive statistics were used to analyze patient demographics and clinic visit characteristics (mean, median, standard deviation [SD], minimum, maximum, frequency, percentage). Two sample t-tests, Chi-square tests, and Fisher’s exact tests were used to compare these descriptive statistics before and after the policy change. Process and wait times were summarized with descriptive statistics pre- and post-policy change. Linear mixed regression models were used to evaluate the effect of the policy change on process and wait times adjusting for appointment type, clinic volume, and daily clinic trends. This model also adjusted for the correlation between visits from the same patient. SAS statistical software (version 9.4, SAS Institute, Cary, NC) was utilized for all statistical analyses.

## Results

### Baseline clinical assessment

Between January 5 and July 3, 2018, a total of 6813 clinical visits occurred at the Kellogg Eye Center glaucoma clinic. Of these, we obtained consent, recorded, and analyzed 1972 (29%) visits from 1589 patients. There were 1031 clinical visits before (52.3%) and 941 visits after (47.7%) the policy change came into effect on March 30, 2018. Table 1 shows a summary of patient demographics and clinical characteristics stratified by visits before and after the policy change. The distributions of patient age (*p* = 0.3), gender (*p* = 0.4), and ethnicity (*p* = 0.4) were not significantly different for visits before and after the policy change, but a significantly smaller percentage of White patients visited before the change (76.7% before versus 81.3% after; *p* = 0.02). Daily patient volume increased after the policy change (mean of 51.9 patients/day before, SD = 16.8, range 5–88; mean of 58.4 patients/day after, SD = 17.4, range 17–95; *p* = 0.04). Additionally, after the policy change a larger percentage of clinic visits were in the morning (56.0% before versus 63.9% after; *p* = 0.0004) and the volume of clinic visits on Wednesdays increased (17% before versus 22.4% after; *p* = 0.003). No significant differences in the distribution of appointment type were observed before and after the policy change (*p* = 0.7).

**Table 1:** Comparison of patient demographics and visit characteristics before and after the policy change

	Before Policy Change (***n*** = 1031 visits, 928 patients, 58 days)			After Policy Change (***n*** = 941 visits, 841 patients, 65 days)			
Continuous Variable	Mean (SD)	Min, Max	Median	Mean (SD)	Min, Max	Median	*P*-value*
Age (year)	65.6 (15.1)	15.9, 102.5	67.2	66.3 (14.1)	13.9, 97.5	68.5	0.33
Daily patient Volume	51.9 (16.8)	5, 88	52	58.4 (17.4)	17, 95	61	0.04
Categorical Variable	frequency (%)			frequency (%)			*P*-value**
Gender							
Male	400 (43.1)			379 (45.1)			0.39
Female	528 (56.9)			461 (54.9)			
Race							
White	700 (76.7)			674 (81.3)			0.02
Black	117 (12.8)			102 (12.3)			
Asian	75 (8.2)			41 (5.0)			
Other	21 (2.3)			12 (1.5)			
Ethnicity							
Hispanic	21 (2.5)			14 (1.9)			0.36
Non-Hispanic	807 (97.5)			739 (98.1)			
Appt Type							
New Patient	94 (9.1)			93 (9.9)			0.69
Return Visit	498 (48.3)			431 (45.8)			
Visual Field	314 (30.5)			284 (30.2)			
Post-op Visit	84 (8.2)			84 (8.9)			
Laser Treatment	39 (3.8)			44 (4.7)			
Urgent	2 (0.2)			3 (0.3)			
Appt Time							
AM	577 (56.0)			601 (63.9)			0.0004
PM	454 (44.0)			340 (36.1)			
Appt Day							
Monday	171 (16.6)			114 (12.1)			0.003
Tuesday	276 (26.8)			269 (28.6)			
Wednesday	175 (17.0)			211 (22.4)			
Thursday	199 (19.3)			161 (17.1)			
Friday	210 (20.4)			186 (19.8)			

*Abbreviations*: *SD* Standard deviation, *Min* Minimum, *Max* Maximum, *Appt* Appointment

^*^2-sample t-test

^**^Chi-square test or Fisher’s exact test (when cell counts < 5)

### Process and wait times

Overall process time significantly decreased (− 4.1 minutes, SE = 1.3, *p* = 0.002) from an average of 44.7 minutes (SD = 27.6) before the policy change to 40.7 minutes (SD = 27.2) after. Descriptive statistics on process and wait times for clinic visits before and after the policy change are displayed in Table 2. A decrease in mean technician process time was observed, from 23.8 minutes before the policy change (SD = 13.6) to 20.8 minutes after the policy change (SD = 12.9). After adjusting for factors that were significantly different before and after the policy change (daily patient volume, time of visit, weekday of visit, and patient race), difference in technician processing time remained similar (2.9 minutes, standard error, SE = 1.3; *p* < 0.0001; Table 3). After adjustment, physician process time showed a non-significant change from a mean of 17.7 minutes (SD = 13.7) before the policy change to 16.7 minutes (SD = 13.8) (*p* = 0.06). Visual field processing time was similar before (mean 22.5 minutes, SD = 14.5) and after (mean 22.1 minutes, SD = 14.7) the policy change (*p* = 0.8).

**Table 2:** Descriptive statistics of process times and wait times during a glaucoma clinic visit, before and after the policy change

	Before Policy Change (***n*** = 1031 visits)		After Policy Change (***n*** = 941 visits)	
Clinic Visit Times (minutes)	Mean (SD)	Median	Mean (SD)	Median
Total Process time	44.7 (27.6)	39	40.7 (27.2)	34
Technician Process time	23.8 (13.6)	21	20.8 (12.9)	19
Physician Process time	17.7 (13.7)	14	16.7 (13.8)	12
Visual Field Process time	22.5 (14.5)	20	22.1 (14.7)	20
Total Wait time	46.0 (32.8)	39	49.9 (33.3)	44
Reception Wait time	11.2 (15.8)	6	13.4 (16.2)	9
In-Process Wait time	15.4 (19.4)	8	16.2 (18.9)	10
Exam Room Wait time	19.5 (21.8)	13	20.3 (21.6)	14
Total Visit time	122 (59)	111	126 (61)	117

*Abbreviations*: *SD* Standard deviation

**Table 3:** Linear mixed regression model results for the effect of the policy change on process times and wait times

Outcome	Estimate^a^ (SE)	*P*-value
Total Process time	−4.1 (1.3)	0.002
Technician Process time	−2.9 (0.7)	< 0.0001
Physician Process time	−1.4 (0.7)	0.06
Visual Field Process time	0.3 (1.1)	0.77
Total Wait time	2.1 (1.6)	0.18
Reception Wait time	0.8 (0.8)	0.27
In-Process Wait time	0.5 (0.9)	0.59
Exam Room Wait time	0.8 (1.0)	0.44
Total Visit time	0.6 (2.9)	0.83

*Abbreviation*: *SE* Standard error

^a^Models were adjusted for daily patient volume, time of visit (AM/PM), weekday, and patient race

There were no significant differences in wait times before and after the policy change (Tables 2 and 3). Total visit wait time was on average 46.0 minutes (SD = 32.8) before the policy change and 49.9 minutes after (SD = 33.3), for an estimated adjusted increase of 2.1 minutes (SE = 1.6; *p* = 0.2). Similarly, mean reception wait time (11.2 minutes before versus 13.4 minutes after), in-process wait time (15.4 minutes before versus 16.2 minutes after), and exam room wait time (19.5 minutes before versus 20.3 minutes after) all showed no significant differences from the policy change (adjusted *p* = 0.3, 0.6, and 0.4, respectively).

Overall total visit time, from patient check-in until check-out, also showed no significant difference before and after the policy change (mean of 122 minutes before [SD = 59] versus 126 minutes after [SD = 61]; estimated adjusted increase of 0.6 minutes [SE = 2.9; *p* = 0.8]).

## Discussion

In this study, we used an RFID-based RTLS, to analyze the impact of a refraction policy change on a tertiary glaucoma clinic’s process and wait times. Evaluation of 1031 patient visits before and 941 visits after the refraction policy change showed the policy change was able to improve technician efficiency. The new policy was associated with decreased technician process time by 2.9 min per patient (*p* < 0.0001) and increased clinic volume by 7 patients per day (*p* < 0.038), with no significant increase in total wait time or total visit time. The automated RTLS system enabled assessment of a lean strategy-guided policy change at each step of clinic flow to fully understand the impact on each aspect of the work flow.

The policy change had a significant effect on clinic process times and the clinic was able to see more patients without increasing wait times. Prior to the policy change, an average of 52 patients were seen daily at the clinic. Since the policy change, there was an associated 2.9 minutes per patient saved in technician processing time, the average amount of time saved per day was approximately 156 minutes. If the time savings was in fact due to the policy change and not due to confounding, technicians could see an additional 7.4 patients daily (156 minutes ±21 minutes/patient = 7.4 patients).

Lean strategy guided quality improvement initiatives can improve efficiency and flow in ophthalmology clinics. For example, in other studies that have used lean methodology: (1) a vitreoretinal clinic moved Optical Coherence Tomography machines from central photography suites to individual examination rooms and saw a 36 and 74% decrease in total visit and wait times respectively [21], and (2) an outpatient retina clinic achieved an 18% faster patient flow time by placing patients into one of five pre-identified common clinic flow paths, adjusting staffing, and optimizing scheduling around derived predictors of patient flow times [3].

Without an accurate, automated, and inexpensive RTLS system, measuring the impact of lean strategy guided policy changes on clinic flow would have been laborious and costly. The RTLS was able to collect data of 1972 visits from 1589 patients over a 6-month period at a capital cost of $31,728 and an estimated recurring expense of $318 for supplies. In comparison, the capital costs for direct observation methods would have been approximately $20 per observer. However, assuming clinic hours of 7 am to 7 pm for a 5-day week, conducting direct observation over 6 months would have required 1440 hours of manpower (60 hours/ week × 4 weeks/ month × 6 months = 1440 hours). At the University of Michigan’s minimum wage for employees (plus 40% fringe benefits) the cost of employing one person to conduct the study would have been $29,400 (1400 hours × $15/ hour × 140% = $29,400). Given that the RTLS was able to capture process and wait times for 23 different clinical spaces, we estimate that we would need a minimum of 6 full time observers placed strategically throughout the clinic to collect data at this level of granularity, with a resultant total study cost of at least $176,000.

Our team designed a passive UHF RFID-based RTLS time-motion capture system because of the low cost per tag ($0.04 - $1.50) and localization range (20 m) sufficient for clinical spaces [19]. However, RTLS systems can be constructed with a myriad of underlying technologies (GPS, Bluetooth, machine vision, ultrasound, infrared, and cellular data). Baslyman et al. [22] used Wi-Fi triangulation and networked infrared hand sanitizer dispensers to track health care provider location and hand hygiene policy adherence in a quality improvement effort at The Ottawa Hospital. An Ultrasound/Wi-Fi hybrid RTLS system has found a niche in assisted living homes, replacing conventional call light systems, as each patient can toggle a button on their ultrasound tag, sharing their precise location with staff and notifying the care providers assigned to that area [23]. This system utilizes ultrasound hubs strategically placed around the medical facility to collect data from tagged staff, patients, and equipment and then Wi-Fi relays the information to a care management mobile app.

In a similar study, the Wilmer Eye Institute General Eye Services Clinic used an infrared-RFID based RTLS to assess a variety of changes following a lean evaluation. Their analysis found that, after the changes, patients spent more time in clinic (99.3 to 112.8 min), but less time with providers (15.4 to 12.1 min with optometrists; 8.3 to 5.8 min with ophthalmologists) [22]. During this same time period, the clinic patient surveys showed a statistically significant improvement in perceived wait time (36.2 to 25.8 min, *p* < 0.001) even though this was not objectively true. RTLS enabled both Kellogg and Wilmer clinics to objectively assess the impact the respective policy changes had on clinic flow.

RTLS systems can easily be modified to meet the unique and diverse needs of different clinics. Vakili and colleagues compared a commercial RTLS system utilizing a battery-powered active infrared tag versus a RFID monitoring modality in an ambulatory ophthalmology clinic and found that a custom RFID RTLS was as accurate as the commercial system, but at one-tenth of the cost [24]. However, our experience tells us that this finding may not be applicable to non-academic settings as the labor cost of our RTLS system was substantially cheaper thanks to talented and knowledgeable student teams volunteering to lead its implementation and customization. Clinics that require sustained technological support and possess the financial means to do so may prefer to buy an infrared RTLS system that is available commercially.

To our knowledge, this was the first study to use lean principles and an RFID-based RTLS in a glaucoma clinic. This combinatory approach has been frequently used in other specialties including in emergency medicine [25], family medicine [26], radiology [27], oncology [28, 29], surgery [30], and intensive care units [31]. It possesses promising potential to guide data-driven change within the field of glaucoma care.

There were limitations both to the RTLS system and to this study. This study was conducted at a single academic sub-specialty practice and the results may not be generalizable to other non-academic or non-glaucoma practices. The RTLS system relies on patients and staff to consistently wear their ID tags and can be influenced by adjacent interference signals. We attempted to mitigate this interference via Hidden Markov Modeling, however, we still needed to exclude data from one exam room due to persistent adjacent-room interference. Additionally, there were time intervals when the RTLS did not register any patient or provider data, most likely due to technological limitations inherent to radiofrequency signals [19], resulting in excluding some participants from the study and an overall system accuracy of 80.6% for patient location and 79.1% for provider location compared to direct observations. Further system improvement could include installing additional RTLS receivers in low signal areas or portions of the eye center that were not monitored by the RTLS (i.e. photography and ultrasound suites). Lastly, this study was conducted over a six-month period and thus may be subject to temporal trends and even changes in technician skill level affecting process times.

## Conclusion

RTLS was shown to be effective at automating time-motion studies and allows objective evaluation of lean informed operations changes. Future studies will focus on clinic flow simulations, optimized scheduling, and iterative clinical operations changes made with the objective of improving efficiency and patient wait times.

## Data Availability

The datasets used and/or analyzed during the current study contains protected health information. As such any interested parties should contact the corresponding author to sign a Data Use Agreement to access the dataset.

## References

[1] Cusumano MA (1988). Manufacturing innovation: lessons from the Japanese auto industry. Sloan Manag Rev.

[2] Poksinska B (2010). The current state of lean implementation in health care: literature review. Qual Manag Health Care.

[3] Ciulla TA, Tatikonda MV, ElMaraghi YA (2018). Lean six sigma techniques to improve ophthalmology clinic efficiency. Retina..

[4] Hribar MR, Biermann D, Read-Brown S (2016). Clinic workflow simulations using secondary EHR data.

[5] Patton MW (2011). Developing a time and motion study for a lean healthcare environment.

[6] Sinsky C, Colligan L, Li L (2016). Allocation of physician time in ambulatory practice: a time and motion study in 4 specialties. Ann Intern Med.

[7] Jones TL, Schlegel C (2014). Can real time location system technology (RTLS) provide useful estimates of time use by nursing personnel?. Res Nurs Health.

[8] Bratt JH, Foreit J, Chen PL, West C, Janowitz B, de Vargas T (1999). A comparison of four approaches for measuring clinician time use. Health Policy Plan.

[9] Sur H, Hayran O, Yildirim C, Mumcu G (2004). Patient satisfaction in dental outpatient clinics in Turkey. Croat Med J.

[10] Duska LR, Mueller J, Lothamer H, Pelkofski EB, Novicoff WM (2015). Lean methodology improves efficiency in outpatient academic gynecologic oncology clinics. Gynecol Oncol.

[11] Waters S, Edmondston SJ, Yates PJ, Gucciardi DF (2016). Identification of factors influencing patient satisfaction with orthopaedic outpatient clinic consultation: a qualitative study. Man Ther.

[12] Thomas S, Glynne-Jones R, Chait I (1997). Is it worth the wait? A survey of patients' satisfaction with an oncology outpatient clinic. Eur J Cancer Care.

[13] Gourdji I, McVey L, Loiselle C (2003). Patients’ satisfaction and importance ratings of quality in an outpatient oncology center. J Nurs Care Qual.

[14] Probst JC, Greenhouse DL, Selassie AW (1997). Patient and physician satisfaction with an outpatient care visit. J Fam Pract.

[15] Rule A, Chiang MF, Hribar MR (2020). Using electronic health record audit logs to study clinical activity: a systematic review of aims, measures, and methods. J Am Med Inform Assoc.

[16] Goldstein IH, Hribar MR, Reznick LG, Chiang MF (2018). Analysis of total time requirements of electronic health record use by ophthalmologists using secondary EHR data.

[17] Holbrook A, Glenn H, Mahmood R, Cai Q, Kang J, Duszak R (2016). Shorter perceived outpatient MRI wait times associated with higher patient satisfaction. J Am Coll Radiol.

[18] McMullen M, Netland PA (2013). Wait time as a driver of overall patient satisfaction in an ophthalmology clinic. Clin Ophthalmol.

[19] Newman-Casey PA, Musser J, Niziol LM, Shedden K, Burke D, Cohn A (2020). Designing and validating a low-cost real time locating system to continuously assess patient wait times. J Biomed Inform.

[20] Newman-Casey PA, Musser JA, Niziol LM (2019). Integrating patient education into the Glaucoma clinical encounter: a lean analysis. J Glaucoma.

[21] Callaway NF, Park JH, Maya-Silva J, Leng T (2016). Thinking lean: improving vitreoretinal clinic efficiency by decentralizing optical coherence tomography. Retina..

[22] Singman EL, Haberman CV, Appelbaum J (2015). Electronic tracking of patients in an outpatient ophthalmology clinic to improve efficient flow: a feasibility analysis and benchmarking study. Qual Manag Health Care.

[23] Baslyman M, Rezaee R, Amyot D, Mouttham A, Chreyh R, Geiger G (2014). Towards an RTLS-based hand hygiene notification system. Proc Comput Sci.

[24] Vakili S, Pandit R, Singman EL, Appelbaum J, Boland MV (2015). A comparison of commercial and custom-made electronic tracking systems to measure patient flow through an ambulatory clinic. Int J Health Geogr.

[25] Laskowski-Jones L (2012). RTLS solves patient-tracking emergency. Health Manag Technol.

[26] Stahl JE, Drew MA, Leone D, Crowley RS (2011). Measuring process change in primary care using real-time location systems: feasibility and the results of a natural experiment. Technol Health Care.

[27] Shukla N, Keast J, Ceglarek D (2014). Modelling variations in hospital service delivery based on real time locating information. Appl Math Model.

[28] Ewing A, Rogus J, Chintagunta P, Kraus L, Sabol M, Kang H (2017). A systems approach to improving patient flow at UVA Cancer Center using Real-Time Locating System.

[29] Barysauskas CM, Hudgins G, Gill KK (2016). Measuring chemotherapy appointment duration and variation using real-time location systems. J Healthc Qual.

[30] Swedberg C (2009). Bon Secours Richmond finds RFID saves $2 million annually.

[31] Yoo S, Kim S, Kim E, Jung E, Lee K-H, Hwang H (2018). Real-time location system-based asset tracking in the healthcare field: lessons learned from a feasibility study. BMC Med Inform Decis Mak.

